# Evaluating patient and public involvement in health research: from theoretical model to practical workshop

**DOI:** 10.1111/hex.12486

**Published:** 2017-06-30

**Authors:** Andy Gibson, Jo Welsman, Nicky Britten

**Affiliations:** ^1^ Department of Health and Applied Sciences University of the West of England Bristol UK; ^2^ Centre for Biomedical Modelling University of Exeter Exeter UK; ^3^ Institute of Health Research University of Exeter Medical School Exeter UK

**Keywords:** evaluation, health research, knowledge spaces, mapping experiences, public involvement, theoretical framework

## Abstract

**Background:**

There is a growing literature on evaluating aspects of patient and public involvement (PPI). We have suggested that at the core of successful PPI is the dynamic interaction of different forms of knowledge, notably lay and professional. We have developed a four‐dimensional theoretical framework for understanding these interactions.

**Aim:**

We explore the practical utility of the theoretical framework as a tool for mapping and evaluating the experience of PPI in health services research.

**Methods:**

We conducted three workshops with different PPI groups in which participants were invited to map their PPI experiences on wall charts representing the four dimensions of our framework. The language used to describe the four dimensions was modified to make it more accessible to lay audiences. Participants were given sticky notes to indicate their own positions on the different dimensions and to write explanatory comments if they wished. Participants’ responses were then discussed and analysed as a group.

**Results:**

The three groups were distinctive in their mapped responses suggesting different experiences in relation to having a strong or weak voice in their organization, having few or many ways of getting involved, addressing organizational or public concerns and believing that the organization was willing to change or not.

**Discussion:**

The framework has practical utility for mapping and evaluating PPI interactions and is sensitive to differences in PPI experiences within and between different organizations. The workshops enabled participants to reflect collaboratively on their experiences with a view to improving PPI experiences and planning for the future.

## 1 INTRODUCTION

Patient and public involvement (PPI) in health‐care delivery and research is embedded in the policies of the English NHS^1^ and is also a requirement for many UK‐based medical research funding bodies. Public involvement in research can be justified on ethical grounds and on the grounds that it improves the quality of research. In this paper, we focus on the argument that it provides an important additional source of knowledge, different to, but equally important to, scientific or professional knowledge.

Patient and public involvement is an international movement, with comparable initiatives in other countries. In the US, the Patient‐Centered Outcomes Research Institute (PCORI) is a major source of research funding, focused on question generation, patient‐centred clinical effectiveness research and broad dissemination. The Canadian Foundation for Healthcare Improvement has a programme of patient and family engagement, and the Consumer Health Forum in Australia includes consumer‐based research and a strong consumer knowledge base. There are also more targeted interventions such as the European Patients’ Academy of Therapeutic Innovation (EUPATI) which aims to increase the capacity of patient organizations to be effective advocates and advisors in medicines research.

In parallel with this, increasing requirement for PPI has been the development of frameworks and methodologies for its evaluation.^2^, ^3^ Evaluation can, amongst other things, provide an evidence base for what constitutes “quality” PPI,^2^ identify what works for whom in what circumstances,^4^, ^5^ evidence the impact of PPI and facilitate planning for future projects.^6^

In a previous paper, and within the context of a theoretical analysis of the social, cultural and political drivers for PPI in health services research and care, we reviewed some existing models of PPI and suggested that they were too inflexible to adequately conceptualize such a diverse and complex phenomenon as public involvement.^7^ Other authors have highlighted the importance for scientists and professionals to recognize the importance of lay knowledge and expertize in their disciplines, and argued that the interaction of lay and professional expertize has the potential to create a more holistic understanding of complex, contemporary health problems.^8^

These situations, where different forms of knowledge (e.g. public, professional or scientific) interact, have been termed knowledge spaces.^9^ They exist in many spheres where public opinion meets, and may conflict with current scientific and professional views. Examples include the controversy over genetically modified (GM) crops, recent debates about fracking and the role of the National Institute for Clinical Excellence (NICE) in approving new drugs and treatments for use by the NHS.^10^

Based on a judicious reading of Habermas,^11^, ^12^ Bourdieu^13^, ^14^ and Fraser,^15^ we derived a four‐dimensional theoretical framework which, we suggested, described the fundamental elements for successful knowledge exchange, and which could be used for mapping and analysing the quality of the interactions that take place within knowledge spaces. Bourdieu, Fraser and Habermas were chosen because they have explicitly engaged with public political debates beyond academia and developed their ideas in the contexts of these debates.

**Figure 1:**
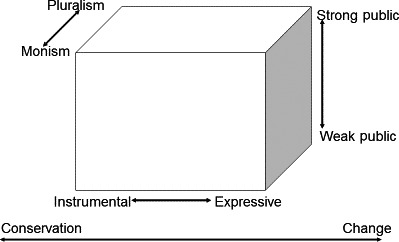
The original theoretical framework (Gibson et al.^7^). Reprinted with permission

The dimensions proposed were expressive to instrumental action, weak to strong publics, monism to pluralism and conservation to change (see Fig. 1). The aim of this framework was to characterize the dynamic and fluid nature of interactions within knowledge spaces, allowing for the fact that individuals or groups can move within these spaces according to their own specific circumstances or nature of the knowledge space.

The paper stimulated discussion about how the framework could be applied in practice. We had suggested that the framework could be used to both plan and evaluate PPI interactions in health services research, but, until recently, we had not taken any steps to do this. Thus, the aim of this paper was to explore the practical utility of the theoretical framework by:

1. describing the evolution of a practical workshop based on the theoretical framework.
2. assessing the suitability of the framework and workshop as tools for mapping and evaluating the experience of PPI within health services research, as seen from the perspective of the public.
3. presenting the results of the workshops from three different PPI groups and discussing how well these capture differences in group characteristics and experiences between and within the groups.

## 2 METHODS

Three workshops were carried out over a period of 6 months with three different PPI groups that provide input to health services research. These groups were selected partly for convenience, but also because, despite some similarities, they all had very different origins, membership, support structures and methods of working. Facilitators were present for group 1 and 3 workshops, but not for group 2. Based on this experience, we have found it helpful to have facilitators involved in the workshop because they can provide information and clarification on specific issues that arise in discussions. However, this role needs to be carefully managed so that it facilitates rather than inhibits discussions.

### 2.1 Group 1

This group was set up approximately six years ago and consists of around 15 people with lived experience of long‐term physical and/or mental health illness, either personally or as a carer. The group is attached to an academic institution in the south‐west of England. Members are offered generic training in evidence‐based medicine. Specific projects have also offered additional training if needed, for example in analysing data. Members are recruited via word of mouth or from open community‐based public workshops. The group meets a minimum of four times a year. Existing members of the group select new members from those who volunteer as vacancies arise, with the aim of being as inclusive as possible. Some members have been longstanding, but there has been a regular turnover of other members. The group is supported by dedicated academic and administrative staff, and two lay members act as business and membership secretaries. Members of the group take it in turns to chair the business meetings. The group has many involvement roles within their host organization including providing a public and patient perspective in research studies, getting involved in teaching activities and in the running of their host organization, for example through representation at management board level and participating in research agenda setting. Members are reimbursed for travel expenses and, in addition, receive “participation payments” for all activities at set rates per half day.

### 2.2 Group 2

Group 2 consists of parents of disabled children and was drawn from a much larger group of approximately 300 members, signed up to an email list. This larger group has been in existence for over five years and provides a public perspective to research into childhood neurological disabilities. The group is attached to an academic institution in the south‐west of England. Training and support has been offered to the group, for example on the social model of disability. Members self‐select, via email, the research opportunities they wish to get involved with, and there is a core of about 20 people who regularly respond to these invitations. Day‐to‐day, the group is supported by an involvement coordinator and overseen by an academic member of staff who chairs the biannual meetings of the open patient advisory group. Families attending meetings are reimbursed for travel expenses and are also paid a fixed rate “participation payment” for their time and commitment.

### 2.3 Group 3

This group has a membership of approximately nine people who are recruited via word of mouth. They were set up approximately 10 years ago and have enjoyed a relatively stable membership with several long‐term members. The group is based in South Yorkshire. Their primary role is to support the work of their local NHS Hospital, to which they are attached, but have increasingly been providing PPI support to academic‐led health services research. They have not been provided with additional training to carry out this role. Some of the members have long‐term health conditions, and most have experience of being patients, or having family members who have been cared for, within the local health‐care system. Members receive expenses to cover travel and lunch and refreshments at meetings, but do not receive a “participation payment.”

The group meets on an ad hoc basis, generally when there is a request for their involvement which happens every 2–3 months. The group is supported part‐time by a member of NHS staff and an academic.

### 2.4 Workshop format

We carried out one‐three‐hour workshop with each of these three groups. They began with a 30‐minute introduction to the framework and its origins which included time for questions and answers. The participants were then invited to think about their personal experiences of involvement as members of a public group within their parent organizations and map these along the four dimensions. Participants in group 1 were also invited to map their experiences of being involved in specific research projects as PPI representatives. It was made clear to participants that not responding to one or more of the dimensions was also acceptable. This activity lasted approximately 1 hour. The final part of the workshop was spent discussing and interpreting the results of the workshop. Each dimension was taken in turn, and participants were asked for any comments or reflections, then a general discussion was held about the group's responses and future directions. This approach was designed to allow individuals to express their individual perspectives as well as contribute to a group discussion. We were also keen to capture participants’ responses to the workshop and receive feedback on what worked well and what could be improved.

In preparing for the first workshop, there were two key challenges. The first was how to present the four‐dimensional framework in an accessible format to a lay audience. In the paper, we had described the four dimensions as: expressive to instrumental action, weak to strong publics, monism to pluralism and conservation to change.^2^ We soon realized that this language was a barrier to using the framework with public groups. We therefore had to think carefully about what we were trying to convey to our participants, and how to take complex pieces of social theory and condense them in to a few clearly understandable sentences.

We made some changes to the wording used on the dimensions of our framework in preparation for the first workshop. However, participants still experienced difficulty understanding the language. As a result, the language used in the workshops passed through iterative stages involving both us and workshop participants, to ensure that we expressed ourselves clearly while staying faithful to the original concepts. We were also aware that this iterative stage would need a definite end point. We would need to standardize the language used in the workshops as much as possible if we wished to compare results from workshops either between groups or within groups over time. We feel that this process of development was completed by the end of the third workshop described here, although there will always be the scope for minor adaptations to suit different contexts.

In some cases, the final language used required minimal alterations from the original, for example “weak to strong publics” became “weak voice to strong voice.” In other cases, the rewording was more radical, for example “monism to pluralism” became “one way to be involved to many ways to be involved.” In the case of one dimension, we had to return to the original underlying concept and rethink what we were trying to express. The dimension “expressive to instrumental action” was originally based on Habermas’^11^, ^12^ distinction between communicative and strategic action. Habermas sees these different modes of action as being characteristic of two distinct social spheres within society, “lifeworld” (characterized by expressive action) and “system” (characterized by instrumental action). In the final version of the framework, this became a continuum from “public concerns to organizational concerns.” This way of explaining this dimension is more accessible to a lay audience and is arguably closer to the underlying distinction between lifeworld concerns and system concerns.

The final dimension “organizational change to organizational inertia” was initially phrased as “change/no change,” but after feedback and discussion, this was replaced with “organization changes to organization resists change” to reflect an organization's willingness to engage with change where appropriate. We have summarized the theoretical backgrounds to the four dimensions in Table 1. A brief verbal lay summary of these was given to workshop participants as part of the introduction to the workshop.

**Table 1:** Theoretical background to the framework

Original dimension descriptor	Workshop descriptor	Theoretical background to dimension
Weak public/strong public	Weak voice/strong voice	Fraser^15^ suggests that not all public voices have an equal ability to influence decision‐making. She makes a distinction between “strong” and “weak” publics. A strong public is one where not only discussions take place, but can also influence decision‐making. This may occur through having access to an organization's decision‐making bodies or being able to bring pressure to bear on them.^12^ Weak publics may discuss issues, but have little chance of influencing decision‐making.
Monism/pluralism	One way to be involved/many ways to be involved	Bourdieu's work on different forms of cultural capital alerted us to the potential for knowledge to take on different forms (e.g. abstract and conceptual or concrete and experiential), but also that these forms may not be equally valued. Furthermore, as Fraser^15^ suggests, channelling diverse cultural forms of expression through a single involvement approach is likely to perpetuate inequality, as any single method is liable to privilege one social or cultural group over another.
Instrumental/Expressive	Organization's concerns/public concerns	This dimension draws on Habermas’ “lifeworld/system” distinction.^6^ “Lifeworld” refers to the contexts of social action, including public opinion, norms and values, as well as individual experiences and behaviours. The “system”, provides the means for the material reproduction of society, for example bureaucracies and markets. The system is characterized by instrumental action, whereas the lifeworld is characterized by more expressive action. Although this distinction can be drawn too sharply, it helps to understand the interface between, for example, the “system” of organizations and the “lifeworld” concerns of patients and public.
Conservation/Change	Organization changes/organization resists change	The degree to which decision‐makers are willing or able to respond to issues raised by participants in knowledge spaces is important. It depends on a number of contextual factors, such as economic resources and national policies.

**Figure 2:**
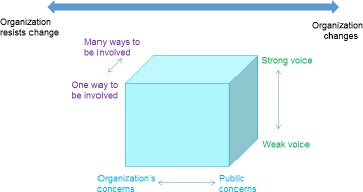
The revised “cube” with alternative terminology

We also realized that the introduction to the workshop needed a diagram of the theoretical framework that reflected this change in language. Participants in the first workshop were confused by the different terminology used in the original cube diagram and their own paperwork and wall chart. The revised cube is presented in Fig. 2.

**Figure 3:**
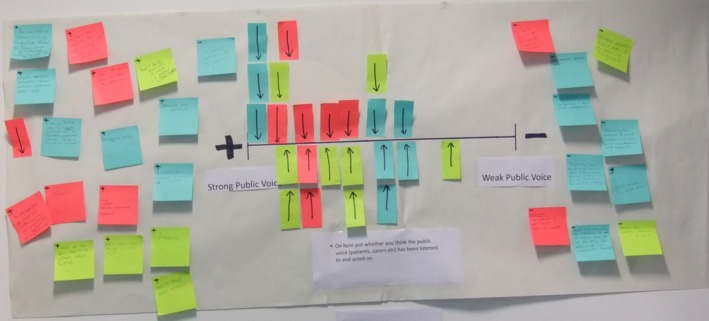
Example wall chart from initial workshop showing the dimension Strong to Weak Voice

The second challenge was how to present the framework in a workshop which allowed participants to use the dimensions to map their own experiences. A method was developed whereby each dimension was separately represented on a wall chart with a short explanatory note reminding participants what was being asked (see Fig. 3). Each participant was given a pack which included sticky notes, and a pen. Participants were asked to use a sticky note with an arrow on it to indicate where along the dimension they felt best represented their own personal PPI experience. People were also invited to write comments on other sticky notes explaining or supporting their arrow placement (illustrated in Fig. 3).

In the first workshop with group 1, we discussed how far participants should exchange their views of their involvement experiences before carrying out the mapping exercise. Participants felt it important to capture, as far as possible, individual subjective experiences in the first instance, with any group discussion and comments left to the end of the workshop. This helped ensure that diverse individual experiences were not lost within a broader group perspective. Although we cannot be sure that people's responses were not affected by group dynamics, the range of results obtained in the workshops suggests that participants did feel able to express their individual perspectives.

Other modifications to the workshop included dropping positive (+) and negative (−) symbols which originally were placed at each end of the dimension. These were intended to indicate “more” or “less” of whatever the axis described, for example towards a stronger voice (+) or towards a weaker voice (−). Similarly, people had been asked to write a positive or a negative sign on their comments to denote whether the comment was supporting a view that there was more or less of something. However, in discussion with participants, it appeared that this could be taken to mean, in the example given, that a strong voice is always a positive, that is the more desirable outcome, which was certainly not the original intention. To prevent this misinterpretation, these positive and negative symbols were removed from both the dimensions and sticky notes in later workshops.

In response to requests from participants, we also added tick marks to divide the dimension into 10 equal spaces. These were not intended to give the impression of a numerical scale, but were to help people gauge their experiences. The resultant maps are visualisations of peoples’ subjective experiences, not numerical scales.

### 2.5 Mapping the workshop data into a diagram

For the purposes of the workshop, the theoretical model presented in our earlier paper^7^ was deconstructed and the four dimensions presented separately for mapping responses and discussion. The challenge was how to collate the participants’ responses and present the information in a way that facilitated interpretation of the groups’ overall responses within the theoretical framework.

**Figure 4:**
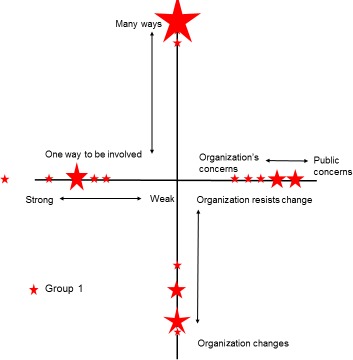
Results from group 1: involvement in organisation

After some consideration of alternatives, a simple crosshair design was felt to provide the simplest, most accurate and easily interpretable method of presenting the workshop data (see Fig. 4). This design enabled data from all four dimensions to be plotted in one diagram with easy visual reference between them. The alignment of each “arm” was selected such that responses clustered around the centre of the cross represent a group with a weak voice, limited ways to be involved, little consideration of public concerns and limited opportunities for organizational change. Conversely, responses towards the extremities of the cross represent a group with a stronger voice and perceived ability to exert organizational change and so on. Use of this design also allowed participants’ responses to be transposed accurately from workshop materials to summary diagram. Where more than one person rated a dimension at the same point, the size of the symbol used was simply increased on a one‐to‐one basis, that is if for one person a symbol size of 0.5 point was used, the symbol representing three people's responses was 1.5 point.

We did not seek research ethics approval to carry out this work. Ethical approval is not normally required to carry out PPI in research.^16^ Exceptions include where PPI representatives are involved in the collection of data. However, these exceptions do not apply to our work. The work described in this paper concerns the development of a workshop designed to facilitate PPI professionals and representatives to reflect on and improve their practice. However, workshop participants were informed that the findings from the workshop would be written up and reported.

## 3 RESULTS

**Figure 5:**
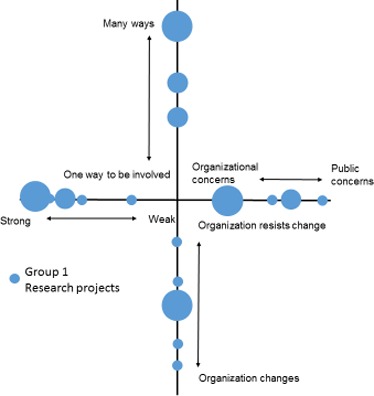
Results from group 1: involvement in research projects

The first “cube” workshop was conducted with ten members of group 1. This group completed the charts twice, once to reflect on their involvement as a PPI group within an organization and a second time to rate their experiences of involvement in individual research studies. Figures 4 and 5 summarize the results of both of these activities for group 1.

Immediately evident in Fig. 4 was that the responses from group 1 tended to cluster towards the extremities of the four dimensions. There was some spread, but responses still remained in the outer half of each dimension. This suggests a group who, collectively, feel they have a strong voice within their parent organization, feel that there are many different ways of getting involved and who believe that the organization is both receptive to change, and willing to change, in response to public concerns. This view was reflected in some of the qualitative comments on the sticky notes posted alongside the chart. For example, in support of ratings of a “strong” public voice…*“*We have the capacity to take our issues forward and do get heard” and similarly, *“*…members do have an influence on the progress of research studies.” But, conversely, one member reported a more negative experience; “Sometimes: not listened to, not heard, taken for granted.”

For the dimension “one way to be involved vs many ways to be involved,” comments were overwhelmingly positive: “Amazing breadth of involvement” and “different types of involvement, rich learning curve.” However, the one negative comment noted “Sometimes over‐whelmed with so many projects and information. Sometimes don't feel appreciated.”

With regard to the dimension “public to organizational concerns,” comments were more evenly balanced: “Nearly always feel my concerns/public concerns are listened to and passed to (the) organisation” vs “The organisation is funded to achieve particular aims which need to be addressed.”

On the whole, participants in group 1 felt that they had a strong voice within a variety of research projects. Comments posted seemed to confirm this “Feel I have been able to make a positive contribution on all occasions.” However, despite the arrow placements, some negative experiences were posted “taken for granted – tokenism or worse.” Responses to the “one way vs many ways to be involved” dimension were also grouped in the outer half of the dimension suggesting various options for participation in research projects. However, the comments varied from “I have had an opportunity to design my own research” to “most researchers want to control involvement.” Regarding “organization's concerns/public concerns,” it was clear that group 1 members felt a strong organizational directive, and similarly for the final dimension the “organization changes/organization resists change,” experiences were clearly diverse with a cluster at the centre point. Fewer qualitative comments on these dimensions were posted but those that were reflected these positions, “Some projects seem fixed and stuck” vs “some projects have been very responsive.”

**Figure 6:**
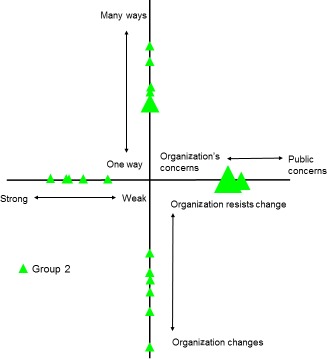
Results from group 2: involvement in organisation

Figure 6 summarizes the results from the workshop with group 2. Their results suggested that members were more diverse in their experiences than group 1. Responses were generally more spread with any clustering occurring at the mid‐point of the relevant dimension. For the dimension “Strong to weak voice,” comments reflected this spread “Definitely feel that my view and views of my family are being heard. Nice to be remembered and called by my first name when communicating” compared to “Not sure how to measure how strong my voice is………” For the dimension “Many ways vs one way to be involved,” more participants mapped around the mid‐point of the dimension with comments such as “If cannot attend meeting how about offering: telephone conferencing or a list before hand of the things being discussed so people can email, write, call you with their point of view?” Other participants were more positive “I think that face to face meetings are a good idea as I can meet up with other families in the region.” Clustering of responses around the mid‐point was most notable for the dimension “Organization's concerns to public concerns.” *Comments* stated: “It is positive that (the organisation) makes strong efforts to accommodate and facilitate public involvement in as many areas as they find practical,” but more negatively, “I sometimes feel guided by the content of the session.” The final dimension “organization changes to organization resists change” produced the most diverse responses, but these responses were in the outer half of the dimension suggesting a tendency towards a perceived willingness to change rather than resistance to change. For some participants, their uncertainty simply reflected how long they had been part of the group. “I am still new to the organization so some of the workshop is ongoing. Not ready yet – don't know outcome – if change will happen.” However, another comment noted that the “Impact of any changes not communicated very well.”

**Figure 7:**
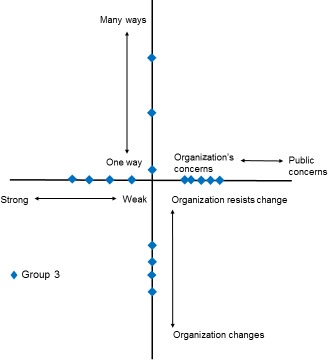
Results from group 3: involvement in organisation

With five participants, group 3 was the smallest workshop. The map of results in Fig. 7 tends to suggest that responses were spread, most notably for the dimension “Single to Multiple,” and tended to sit between the mid‐point and the central meeting point of the four dimensions. Comments reflected these ratings. For example, regarding “strong to weak voice,” participants noted “For strong public voice consumers need education and training” and “I think there are the facilities to listen to the concerns, But they (the public) have to have the chance.” In respect of “one way to be involved vs many ways to be involved,” the following comments were received…. “Involvement multiple types but are the right people involved?” and “I am not quite sure about this and so I've erred on the negative side.” The map also suggests a clear perception that the organization's concerns take precedence over public concerns with comments to this effect: “(The organization's) view dominates, lack of feedback.” The mapping of responses on “organization changes to organization resists change” spread over the centre of the dimension with comments reflecting some perceived inertia to change: “Think there is potential to change. They do listen but process is slow,” or a simple lack of knowledge “Not been involved long enough, to have any feedback on change.”

Importantly, the maps from the workshops, taken together with the posted comments, provide a clear and immediate picture of participants’ subjective experiences of involvement and some indication of the direction that change might need to take in order to improve these interactions. The general discussion that occupies the last hour of the workshop enables participants to discuss these comments and issues in more depth both with other members of the group and those leading the workshop. To illustrate this process, we noted several themes emerging consistently across the three groups.

Communication and feedback were at the forefront of participant concerns: “Communication is the key,” “Where has our input gone? How can we know how strong/weak it is?”

Issues around different ways to be involved were common. Some participants liked the face‐to‐face style of many PPI meetings, but others noted feeling like they were “missing out if I don't attend.” Participants also sought to further discuss practical solutions such as suggesting that agendas could be sent out in advance of contributions made by phone. An over‐reliance on email was noted with telephone calls preferred by some.

The informal and inclusive format of the workshop enabled participants to speak about the emotional as well as practical aspects of PPI. Participants valued the opportunity to share these experiences “we have space to form bonds and discuss emotion.” But, the discussion also highlighted some professional discomfort for the emotional side of PPI “sometimes researchers find it difficult to deal with the personal.”

The general discussion also highlighted that whereas some participants were aware of some organizational boundaries and constraints “at the end of the day researchers are paid by [the organization] and money for projects is via stakeholders,” others seemed less aware of the demands of a research environment and expressed frustration at the seemingly slow progress of projects and time to publication “Changes take too long—projects take ages to go through and you feel like it won't benefit you or anyone in your lifetime.”

## 4 DISCUSSION

This paper set out to explore the possibility that our theoretical framework may be of practical use as a tool for mapping and evaluating PPI interactions. The findings presented here indicate that the framework was able to identify important differences in the subjective experiences of lay participants across the three groups we have described. Furthermore, these different experiences appear to be related to how these groups are organized and their differing relationships with their parent organizations. Group 1 had been set up at the inception of its parent organization, and the two had developed together. This appears to be reflected in the generally stronger and more clustered responses. The second group, based on a mailing list with a smaller active group, has less clustering, and the responses tend to be closer to the centre of the diagram. Finally, group three's responses are even more closely positioned at the centre of the diagram and are generally more spread, perhaps reflecting this group's more ad hoc involvement in research.

The charts also appear to be sensitive to different activities within the same research organization. The second chart for group 1 (Fig. 5) recorded experiences within specific research projects rather than the organization as a whole and produced a chart with a wider spread of results. The results for group 1 in Fig. 4 suggest a group that feels that it has a strong voice within an organization that is responsive to its concerns offers differing ways to be involved and is willing to change in response to feedback. In contrast, in Fig. 5, the same group feels that the ability of individual research projects to change in response to feedback was much more diverse than in the organization as a whole. Individual projects were also seen as much less responsive towards public concerns with some PPI reported as “taken for granted, tokenism or worse.” These results also highlight the value of the mapping exercise in allowing both the public participants and those leading the PPI to, in real time, identify areas or activities where, despite a strong overall group profile, some specific experiences may give cause for concern and require some discussion with the relevant professionals. This may not have become apparent without the structure of the workshop.

We noted that in all of the workshops, participants were generally less likely to post responses on the “organization changes to organization resists change” dimension. As evidenced by some of the comments, this may simply reflect that new members in a group may not feel that they have sufficient experience to comment on this. However, in discussion, it also became clear that many participants did not feel able to comment because they had not received sufficient or specific feedback that would allow them to assess whether or not the organization had changed in response to their concerns. Non‐responses may therefore be important in helping to highlight a breakdown in PPI interactions and indicate opportunities to develop, where appropriate, more embedded PPI.

Participants in all three workshops expressed the view that the workshops had been challenging but enjoyable. They reflected that the workshop encouraged them to think about their involvement experiences and interactions from a more holistic, long‐term perspective and also in relation to the views of other group members and the workshop or PPI leads. They felt it enabled them to reflect collaboratively upon the future purpose and direction of their own and the group's involvement in the organization. Participants were asked if there were aspects of their involvement experiences that were not covered by the structure and format of the workshops, but none were identified. This suggests that the four dimensions of the framework adequately cover the fundamental elements underpinning PPI.

The fact that the results of the mapping exercise are immediately available to workshop participants, as opposed to sometime later, after a researcher has analysed the material, is also important. The participatory nature of the workshop helped to develop a sense of group cohesion and co‐production which would not have been possible with a more traditional questionnaire or interview‐based approach. Participants reported that they had not previously been given the chance to discuss their involvement in this way and valued the opportunity to do this. These discussions might raise participants’ awareness that in some settings, they work within externally constrained agendas, and to consider ways of shaping these agendas. In this way, the workshops facilitated planning of future involvement activities.

On the basis of these initial findings, we feel cautiously optimistic that our framework could be used to map, plan and evaluate PPI interactions based on information gathered from lay participants. The approach we have described is both participative and orientated to developing positive recommendations for improvement.

### 4.1 Limitations and future directions

Our experience with these workshops indicates that they work well with public involvement groups where participants are actively involved in shared group activities where evaluation is valuable. The workshop creates a space where collective reflection and planning can take place. This may be involvement in a specific research activity, for example research prioritisation or research project, or within a particular organizational context, for example a university department. This workshop can be used in a cross‐sectional way, as described here, to help make useful comparisons between groups in different organizations, or between different involvement activities within a single group. We have also used the workshop longitudinally, to examine changes in PPI interactions across time. It is important that the workshops are run consistently using the same terminology and explanations if comparisons between findings are to be made.

The workshop requires participants to commit half a day to the activity. This might mean that only the more active members of a group become involved. This is not necessarily a drawback, as long as the people carrying out the workshops are aware that other members’ views may not be represented. Some of these difficulties can be overcome by holding the workshops at accessible times and locations, offering to pay expenses such as travel and childcare and reimbursement for the time committed.

Although the workshops described in this paper primarily evaluated PPI interactions from the perspective of public contributors, it would be possible to conduct cube workshops with lay and professional groups separately and then compare the results, highlighting commonalities and areas of divergence in experiences as the basis of developing discussions about the strategic direction of PPI activities within organizations. This is a possibility which we hope to pursue in the future.

It has been suggested that academic papers on PPI treat involvement as an intervention to be evaluated such as any other for evidence of positive impacts, unintended negative side‐effects and cost‐effectiveness.^17^ We feel that it is important when evaluating PPI to keep in mind that PPI is fundamentally about an exchange of knowledge and ideas. This work originated with the concept of knowledge spaces as places where diverse forms of knowledge can interact to create a more holistic understanding of a complex problem.^9^ This is a different perspective from that taken by evaluations which focus on outcomes and impacts.^3^, ^5^ Viewed from the perspective adopted here, a key issue becomes how do we facilitate meaningful interactions between members of the public, academics and other professionals? How can we assess and improve the quality of these interactions and how do we know that these interactions have made a difference? We feel that the theoretical framework we have developed and the practical workshops that we have derived from it provide one solution to answering these questions.
